# Molecular Characteristics of Regional Chromoblastomycosis in Guangdong, China: Epidemiological, Clinical, Antifungal Susceptibility, and Serum Cytokine Profiles of 45 Cases

**DOI:** 10.3389/fcimb.2022.810604

**Published:** 2022-02-18

**Authors:** Hongfang Liu, Jiufeng Sun, Minying Li, Wenying Cai, Yangxia Chen, Yinghui Liu, Huan Huang, Zhenmou Xie, Weiying Zeng, Liyan Xi

**Affiliations:** ^1^ Dermatology Hospital, Southern Medical University, Guangzhou, China; ^2^ Guangdong Dermatology Hospital of Anhui Medical University, Guangzhou, China; ^3^ Guangdong Provincial Institute of Public Health, Guangdong Provincial Center for Disease Control and Prevention, Guangzhou, China; ^4^ Sun Yat-sen Memorial Hospital of Zhongshan University, Guangzhou, China; ^5^ Department of Dermatology and Venerology, Guangzhou First People’s Hospital, Guangzhou, China

**Keywords:** chromoblastomycosis, ITS rDNA, antifungal susceptibility, *Fonsecaea* spp., cytokine

## Abstract

Chromoblastomycosis (CBM) is a chronic disease caused by several species of dematiaceous fungi. In this study, a regional collection of 45 CBM cases was conducted in Guangdong, China, a hyper-endemic area of CBM. Epidemiology findings indicated that the mean age of cases was 61.38 ± 11.20 years, long duration ranged from 3 months to 30 years, and the gender ratio of male to female was 4.6:1. Thirteen cases (29%) declared underlying diseases. Verrucous form was the most common clinical manifestation (*n* = 19, 42%). Forty-five corresponding clinical strains were isolated, and 28 of them (62%) were identified as *F. monophora*; the remaining 17 (38%) were identified as *F. nubica* through ITS rDNA sequence analysis. Antifungal susceptibility tests *in vitro* showed low MICs in azoles (PCZ 0.015–0.25 μg/ml, VCZ 0.015–0.5 μg/ml, and ITZ 0.03–0.5 μg/ml) and TRB (0.015–1 μg/ml). Itraconazole combined with terbinafine was the main therapeutic strategy used for 31 of 45 cases, and 68% (*n* = 21) of them improved or were cured. Cytokine profile assays indicated upregulation of IL-4, IL-7, IL-15, IL-11, and IL-17, while downregulation of IL-1RA, MIP-1β, IL-8, and IL-16 compared to healthy donors (*p* < 0.05). The abnormal cytokine profiles indicated impaired immune response to eliminate fungus in CBM cases, which probably contributed to the chronic duration of this disease. In conclusion, we investigated the molecular epidemiological, clinical, and laboratory characteristics of CBM in Guangdong, China, which may assist further clinical therapy, as well as fundamental pathogenesis studies of CBM.

## Introduction

Chromoblastomycosis (CBM) is a chronic cutaneous and subcutaneous fungal infection disease caused by several species of dematiaceous fungi, and mainly distributed in tropical and subtropical areas worldwide. In 2017, CBM was first recognized as a neglected tropical disease by the World Health Organization (WHO, 2017). The etiological agents of CBM are mainly related to the genera *Cladophialophora*, *Phialophora*, and *Fonsecaea* (Ameen, 2010). Incidence of CBM usually follows a trauma with a contaminated organic material such as plant thorns, wood, plant debris, grass, and tree cortex, leading to the implantation of the fungus in the subcutaneous tissues, where the fungus changes from mycelial form to its parasitic form composed of muriform cells. The muriform cells are the key to CBM development, which are extremely resistant to the harsh conditions imposed by the host immune system (Queiroz-Telles et al., 2017).

*Fonsecaea* are the most common pathogens of CBM (Najafzadeh et al., 2011), including four clinical related species, *F*. *pedrosoi*, *F*. *nubica*, *F*. *monophora*, and *F*. *pugnacius*. Although the morphology of these four species is extremely similar, the pathogenicity is different from each other. *F. pedrosoi* and *F. nubica* appear to be exclusively associated with CBM under skin (De Hoog et al., 2004), whereas *F. monophora* and *F. pugnacius* were found to gain a much wider tissue tropism, e.g., brain, gallbladder and lymph node (Saberi et al., 2003; Surash et al., 2005; de Azevedo et al., 2015). The archived studies indicated that the clinical manifestations of CBM could be classified into 6 types (clinic, nodular, plaque, verrucous, tumor, cicatricial, and mixed form) and three stages (mild, moderate, and severe form) (Queiroz-Telles et al., 2017). The clinic treatment of CBM is quite challenging; there is no universal treatment protocol that could be followed. Many modalities, including antifungals, immunomodulatory therapy, physical methods, photodynamic therapy (PDT), and surgical excision, have been used solely or combined for the treatment of CBM in previous reports (Paniz-Mondolfi et al., 2008; Zhang et al., 2009; Queiroz-Telles and Santos, 2013; de Sousa et al., 2014; Hu et al., 2019; Huang et al., 2019). So far, itraconazole (ITC) and terbinafine (TRB) are the most commonly used antifungal drugs in the treatment of CBM (Lopez and Mendez, 2007; Daboit et al., 2013; Daboit et al., 2014).

The host defense mechanisms in CBM are still poorly understood (Seyedmousavi et al., 2014). Several studies demonstrated that both innate and adaptive immune responses are required for effective containment of infections (Sousa et al., 2011). Initially, Mazo et al. (2005) demonstrated that the severity of CBM correlated with the balance of TH1 and the TH2 immune response. Another study manifested that Treg/Th17 imbalance in CBM patients decreased local immune response to the fungus (Silva et al., 2014). In our previous studies, we found that the melanization of *F. monophora* probably inhibited production of nitric oxide and T helper cell type I cytokines in murine macrophages, and enhanced persistence of the agent in both *in vitro* and *in vivo* (Zhang et al., 2013; Jiang et al., 2018). However, the detailed immune response against fungus remains unclear.

Given the different clinical manifestations of CBM cases, variable causative agents, and poorly understood immune response, there is a great need to explore the real situation in different endemic areas of CBM. In this study, a regional collection of 45 CBM cases was from the Dermatology Hospital of Southern Medical University during 2016 to 2021, aiming to investigate the epidemiology characteristics, evaluate the susceptibility of antifungal agents against isolates *in vitro*, and clarify the cytokine profiles of these CBM cases in Guangdong, China, a hyper-endemic area of CBM.

## Materials and Methods

### Clinical Data of Chromoblastomycosis Cases

All of the 45 diagnosed CBM cases who visited the Dermatology Hospital of Southern Medical University from 2016 to 2021 were enrolled in this study. The detailed clinical data (age, sex, site of predilection, etiologic agent, trauma, underlying disease, time of evolution, severity, lesion type, treatment, and outcome) of CBM cases were collected (**Table S1**, Clinical Data of 45 cases of Chromoblastomycosis ). The procedure associated with patients was approved by the Ethics Committee Board of Dermatology Hospital of Southern Medical University.

### Isolation and Morphology Identification

The skin lesion of each patient was taken and checked under a microscope when they visited our hospital the first time, and then sent for further culture on sabouraud dextrose agar (SDA) at 26°C for 2 weeks. The suspect colonies were taken and morphologically identified under a microscope. The pure cultured isolates were grown at 26°C on both SDA and potato dextrose agar (PDA) plates for 2 to 3 weeks. Slide cultures were done under the same conditions and checked again with microscopy to confirm the morphology.

### Molecular Identification

The morphology-identified pure isolates were cultured again under the above conditions for 2–3 weeks. The fungi were collected (~0.5 g) into a clean microtube (2 ml). The genome DNA was extracted using the protocol adapted from DNA Purification from Yeast (The Gentra Puregene Yeast/Bact. Kit, QIAGEN, Germany). The ITS regions were used for species identification for these isolates. The PCR amplification was performed in T100 Touch Thermal Cycler (Bio-Rad, USA) using primers ITS4 (5′-TCCTCCGCTTATTGATATGC-3′) or ITS1 (TCCGTAGGTGAACCTGCGG) and ITS5 (5′-GGAAGTAAAAGTCGTAACAAGG-3′) in a 50-μl reaction system containing 1 μl of template genome DNA, 25 μl of Taq polymerase (Premix Taq, TaKaRa Taq Version 2.0 plus dye, Japan), 20 μl of ddH_2_O, and 2 μl of each primer (10 μM). PCR was conducted with the following conditions: 95°C for 3 min, followed by 34 cycles of 95°C for 30 s, 55°C for 30 s, and 72°C for 1 min and a final extension of 72°C for 5 min. PCR amplicons were checked using electrophoresis in 1% agarose gel (100 V for 45 min). The positive amplicons were purified and sent to Sangon Biotech (Shanghai, China) for Sanger sequencing as described previously (White et al., 1990; Gauthier, 2007) using the same primers. The homology evaluations were done on the GenBank database using BLAST analysis available at www.ncbi.nlm.nih.gov. All the sequences attained in this study were submitted to the GenBank database under accession numbers listed in **Table S2**, Genbank accession number of 45 clinical strains.

### Phylogenetic Analysis

A phylogenetic method was used to explore the relationships among the 45 clinical isolates. ITS sequences were aligned using MUSCLE with a panel of 50 reference strains from the mycobank database (http://www.mycobank.org), and a maximum likelihood (ML) phylogenetic tree was constructed with MEGA 6.0 using the K2+G model with 500 bootstrap replicates. Bootstrap > 70 were considered as consistent support branches.

### Antifungal Susceptibility Testing

The Sensititre YeastOne^®^ panel trays containing serial twofold dilutions of anidulafungin (AND, 0.015–8 μg/ml), micafungin (MFG, 0.008–8 μg/ml), caspofungin (CAS, 0.008–8 μg/ml), 5-flucytosine (5-FC, 0.06–64 μg/ml), posaconazole (PCZ, 0.008–8 μg/ml), voriconazole (VCZ, 0.008–8 μg/ml), itraconazole (ITR, 0.015–16 μg/ml), fluconazole (FLU, 0.12–256 μg/ml), and amphotericin B (AMB, 0.12-8 μg/ml) were purchased from Thermo Fisher Company (Shanghai, China). The packages of YeastOne^®^ panels were stored at room temperature until testing was performed. A working conidium suspension of approximately 0.5–5 × 10^4^ cells/ml was prepared in YeastOne^®^ broth (Trek). Each well of the dried YeastOne^®^ panels was rehydrated with 100 μl of the working conidium suspension delivered by a multichannel pipetting device. The YeastOne^®^ panels were covered with seal strips and incubated at 35°C for 120–144 h in a non-CO_2_ incubator. The colorimetric MIC endpoints were determined by visual reading. Fungal growth was evident as a color change from blue (no growth) to red (growth). Colorimetric MIC results for all of the testing drugs were defined as the lowest concentration of antifungal agent that prevented the development of a red color from firstly blue or purple. Because terbinafine (TER) is another commonly used antifungal agent against *Fonsecaea* spp. infection, not involved in YeastOne^®^ method, we used the ClSI method to test its susceptibility according to the reference (M38, Reference Method for Broth Dilution Antifungal Susceptibility Testing of Filamentous Fungi, 3rd Edition). The concentration range of terbinafine was 0.015–8 μg/ml. Reference broth microdilution testing was performed exactly as outlined in the CLSI document with a conidium suspension of 1 ×10^4^ CFU/ml in RPMI 1640 medium buffered to pH 7.0 with 0.165 mol/L morpholinepropanesulfonic acid (MOPS) buffer. The panels were incubated at 35°C and observed for the presence or absence of growth at 120–144 h depending on the growth of each fungus.

### Human Serum Cytokine Determination

To evaluate the host immune response to Fonsecaea spp., the serums from 18 of 45 patients and 4 healthy donors were taken before treatment and used for cytokine production screening using protein chip array. The serums of healthy donors were collected from a vaccination project that aimed to collect otherwise healthy donors. The white blood cell, percentage of neutrophil, percentage of lymphocyte, and neutrophil-to-lymphocyte ratio of healthy donors were normal. All protocol of protein chip array reference to Quantibody Human Inflammation Array3 kits to quantitative measurement of 40 human cytokines (RayBiotech, Norcross, GA, USA). This array is a glass slide-based antibody assay that allows us to conduct rapid, accurate expression profiling of 40 human cytokines. Two hundred microliters of each human serum sample was used for this protein chip array. After incubation with serum samples, the cytokines were captured by the antibodies printed on the solid surface of glasses. The slide was blocked, and then washed several times according to the protocol instruction. Then, a second biotin-labeled detection antibody was added to recognize different epitopes of the target cytokines. The signal can be determined through the streptavidin-conjugated Cy3 equivalent dye after scanning by laser fluorescent scanner systems (Axon GenePix, CA, USA). The density of each spot was taken and normalized using Q-analyzer software (no.GSM-CAA-4000-SW).

### Statistical Analysis

GraphPad Prism software (version 8.3.0. San Diego, California USA, www.graphpad.com) was used for all statistical analyses. The difference in clinical severity, lesion types, and species distribution was analyzed using Wilcoxon rank sum test and Fisher’s exact test. The correlation was tested by Spearman test. Antifungal test data among drugs were tested by Mann–Whitney *U* test and cytokine data were tested by unpaired *t*-test. The error bars indicate standard deviation of the mean. The portion results are presented as the mean ± standard deviation; *p* < 0.05 indicated statistical significance.

## Results

### Clinical Findings of Chromoblastomycosis Cases

All of the 45 CBM patients come from various cities of Guangdong province. Their clinical characteristics are listed in **Table 1**. The age ranged from 24 to 86 years old (61.38 ± 11.20). The ratio of male to female was 4.6:1. The occupation of 25 cases (56%) was farmer and 17 cases (38%) reported a trauma history. The course of CBM lasted from 3 months to 30 years, while most of the patients (*n* = 38, 84%) were more than 1 year. Thirteen cases (29%) had underlying diseases, namely, diabetes (*n* = 3), systemic lupus erythema (*n* = 2), nephrotic syndrome (*n* = 1), lung cancer (*n* = 1), chronic colitis and bronchitis (*n* = 1), and cardio-cerebral vascular-related diseases (*n* = 5), and 26 patients (58%) were immunocompetent and denied underlying diseases, while the data of the remaining 7 cases were not available. The most common infection sites occurred in the upper extremity (49%, *n* = 22), followed by the lower extremity (40%, *n* = 18) and trunk (11%, *n* = 5). Verrucous form was the most common clinical presentation for all 45 cases (42%, *n* = 19), followed by plaque form (38%, *n* = 17), cicatricial (7%, *n* = 3), tumorous (2%, *n* = 1), nodular (2%, *n* = 1), and mixed form (9%, *n* = 4) of lesions, which were also noted in this study. The classification of severity of CBM was based on type of lesions, size, and extension [3], including 13 mild cases (29%), 14 moderate cases (31%), and 18 severe cases (40%). Oral itraconazole (200 mg/day) in combination with terbinafine (250mg/day) as the initial antifungal therapy was used in 31 cases (69%) solely or combined with other adjunctive therapy (e.g., photodynamic therapy, surgery, and thermotherapy) for 3–6 months. Whether these patients need to be followed by monotherapy of itraconazole or terbinafine as maintenance treatment depended on their clinical outcomes. Among them, 21 cases (68%) improved or were cured. Three patients who received ALA-PDT (5-Aminolaevulinic acid PDT) combined with antifungal agents improved greatly after 3–4 treatment sessions at an interval of 1 week. The duration of treat time recorded in our hospital ranged from 1 to 37 months (mean 8.62 ± 9.46 m). Twenty-six of 45 CBM cases (58%) showed notable improvement, 3 cases had complete remission, 5 cases had relapse, while 11 cases (24%) were lost to follow-up.

**Table 1:** Clinical data of 45 cases of Chromoblastomycosis.

Subject	Type		Data
Age	Average ± STD		61.38 ± 11.20 (24–86 years)
Gender	Male		82% (37/45)
	Female		18% (8/45)
Etiology agent	*F. monophora*		62% (28/45)
	*F. nubica*		38% (17/45)
Occupation	Farmer		56% (25/45)
	Other		22% (10/45)
	NA		22% (10/45)
Trauma	Y		38% (17/45)
	N		42% (19/45)
	NA		20% (9/45)
Underlying disease	Y		29% (13/45)
	N		58% (26/45)
	NA		13% (6/45)
Duration	Average ± STD		7.10 ± 7.33 years (3 months–30 years)
Sites	Upper extremity		49% (22/45)
	Lower extremity		40% (18/45)
	Trunk		11% (5/45)
Skin lesion	verrucous		42% (19/45)
	Plaque		38% (17/45)
	Cicatricial		7% (3/45)
	Tumorous		2% (1/45)
	Nodular		2% (1/45)
	Mixed form		9% (4/45)
Severity	Mild		29% (13/45)
	Moderate		31% (14/45)
	Severe		40% (18/45)
Treatment	Antifungal agents	ITR+TER	53% (24/45)
		ITR	16% (7/45)
		TER	2% (1/45)
	Combined therapy	ITR+TER+PDT	7% (3/45)
		ITR+TER+Surgery	7% (3/45)
		ITR+Surgery	7% (3/45)
		ITR+TER+Thermotherapy	2% (1/45)
	No treatment		7% (3/45)
Outcome	Cure		7% (3/45)
	Improve		58% (26/45)
	Relapse		11% (5/45)
	Lose follow-up		24% (11/45)
Time of treatment	Months		8.62 ± 9.46 (1–37 months)

NA, not available; ITR, itraconazole; TER, terbinafine; PDT, photodynamic therapy; m, month; y, year.

### Molecular Identification and Phylogenetic Analysis

**Figure 1:**
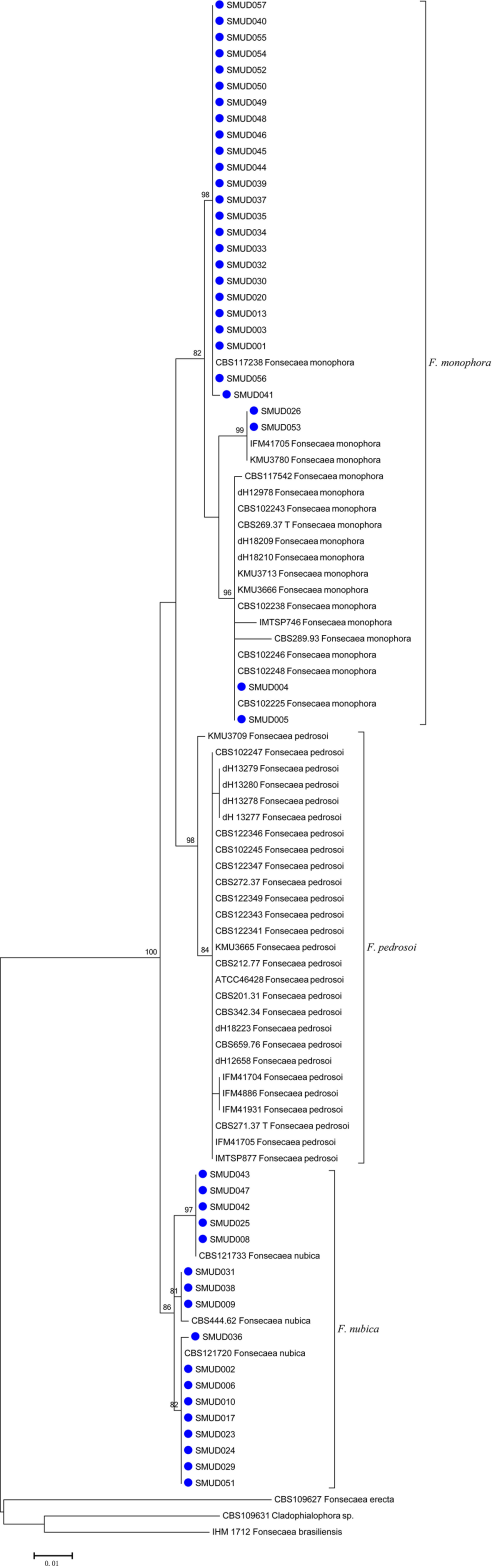
An ITS maximum likelihood (ML) phylogenetic tree of strains isolated in this study. Three species, *F. monophora*, *F. pedrosoi*, and *F. nubica*, were shown in the tree. The tree was constructed with MEGA 6.0 using K2+G model with 500 bootstrap replicates. Bootstrap > 70 were considered as consistent support branches. The strains in this study were marked as a blue dot in tips.

Among the 45 corresponding clinical isolates identified through ITS rDNA sequence analysis, 28 (62%) were *F. monophora*, and the remaining 17 (38%) were *F. nubica*. The ITS sequencing alignment scores of the fungal isolates herein studied exhibited 99%–100% identity compared with corresponding ITS sequences deposited in the mycobank database (**Figure 1**). There were three major clusters observed in the phylogenetic tree, including *F. pedrosoi*, *F. monophora*, and *F. nubica*. *F. erecta* and *F. brasiliensis* were classified as an outgroup in this study, which indicated a variety of evolution history for different species of *Fonsecaea*. The strains in this study were assigned into the cluster of *F. monophora* (*n* = 28) and *F. nubica* (*n* = 17) with bootstrap support, which is consistent with the results of molecular identification.

### Correlation Between Clinical Manifestation and Species Distribution

The correlation of clinical severity and species distribution was analyzed using the Wilcoxon rank sum test. Although statistical results showed that *F. monophora* (*n* = 11) caused a more mild form of CBM than *F. nubica* (*n* = 2), not supported by statistical test (*z* = −1.458, *p* = 0.145). There was no difference and correlation between fungal strains and skin lesion, as well as skin lesion and severity tested by Fisher’s exact test (*p* = 0.846 > 0.05) and Spearman correlation test (*r* = 0.04) (**Table 2**).

**Table 2:** Clinical manifestation and species distribution of 45 CBM cases.

Strain	Severity	Type of lesions						Cases
		Verrucous	Plaque	Nodular	Tumorous	Cicatricial	Mixed	
*F. monophora*	Mild	6	4			1		11
	Moderate	1	5	1				7
	Severe	5	2			1	2	10
*F. nubica*	Mild		1			1		2
	Moderate	2	5					7
	Severe	5	1		1		1	8
Total		19	18	1	1	3	3	45

### Antifungal Susceptibility Testing

The geometric mean of MICs, MIC ranges, MIC50s, and MIC90s for the *Fonsecaea* isolates is summarized in **Table 3**. PCZ (MIC range 0.015–0.25 μg/ml), VCZ (MIC range 0.015–0.5 μg/ml), and ITZ (MIC range 0.03–0.5 μg/ml) were the azole antifungal agents that showed high activity against the *Fonsecaea* isolates. TRB (MIC range 0.015–1 μg/ml) was also active to these strains, but less effective than in azoles. For echinocandins, most isolates showed less sensitivity to AND (MIC range 2–>8 μg/ml), MFG (MIC range 1–>8 μg/ml), and CAS (MIC range 0.25–>8 μg/ml). The susceptibility profile between *F. monophora* and *F. nubica* showed no differences among the antifungal agents (*p* > 0.05).

**Table 3:** Minimal inhibitory concentrations (MIC) of 10 antifungal agents against 45 clinical isolates of *Fonsecaea* spp.

Antifungal	MIC (μg/ml)													
agent	*Fonsecaea* spp.(*n* = 45)				*F. monophora* (*n* = 28)					*F. nubica* (*n* = 17)				
	Range	MIC_50_	MIC_90_	GM	Range	MIC_50_	MIC_90_	GM	Range		MIC_50_	MIC_90_	GM
AND	2–>8	4	>8	3.24	2–>8	2	>8	3.09	2–>8		4	>8	3.50
MFG	1–>8	8	>8	6.35	2–>8	8	>8	6.32	1–>8		8	>8	6.42
CAS	0.25–>8	1	>8	1.74	0.25–>8	1	8	1.48	0.25–>8		2	>8	2.27
5-FC	0.12–8	4	8	5.46	0.25–16	8	8	5.76	0.12–16		4	8	4.96
PCZ	0.015–0.25	0.12	0.25	0.10	0.015–0.25	0.06	0.12	0.10	0.015–0.5		0.12	0.25	0.12
VCZ	0.015–0.5	0.06	0.12	0.09	0.015–0.25	0.06	0.12	0.08	0.015–0.5		0.06	0.12	0.10
ITR	0.03–0.5	0.25	0.25	0.18	0.03–0.25	0.25	0.25	0.18	0.03–0.5		0.12	0.25	0.17
FLU	8–128	32	32	28.62	8–64	32	32	26.00	8–128		32	32	32.94
AMB	1–8	4	8	4.71	1–8	4	8	4.79	1–8		4	8	4.59
TRB	0.015–1	0.25	1	0.35	0.015–1	0.25	1	0.34	0.03–1		0.25	0.5	0.36

MIC refers to the minimal inhibitory concentrations.

The MIC50 and MIC90 values correspond to the minimal inhibitory concentration of the antifungal able to inhibit the growth of 50% and 90% of all fungal isolates, respectively.

GM, Geometrical mean.

### Human Serum Cytokine Determination

**Figure 2:**
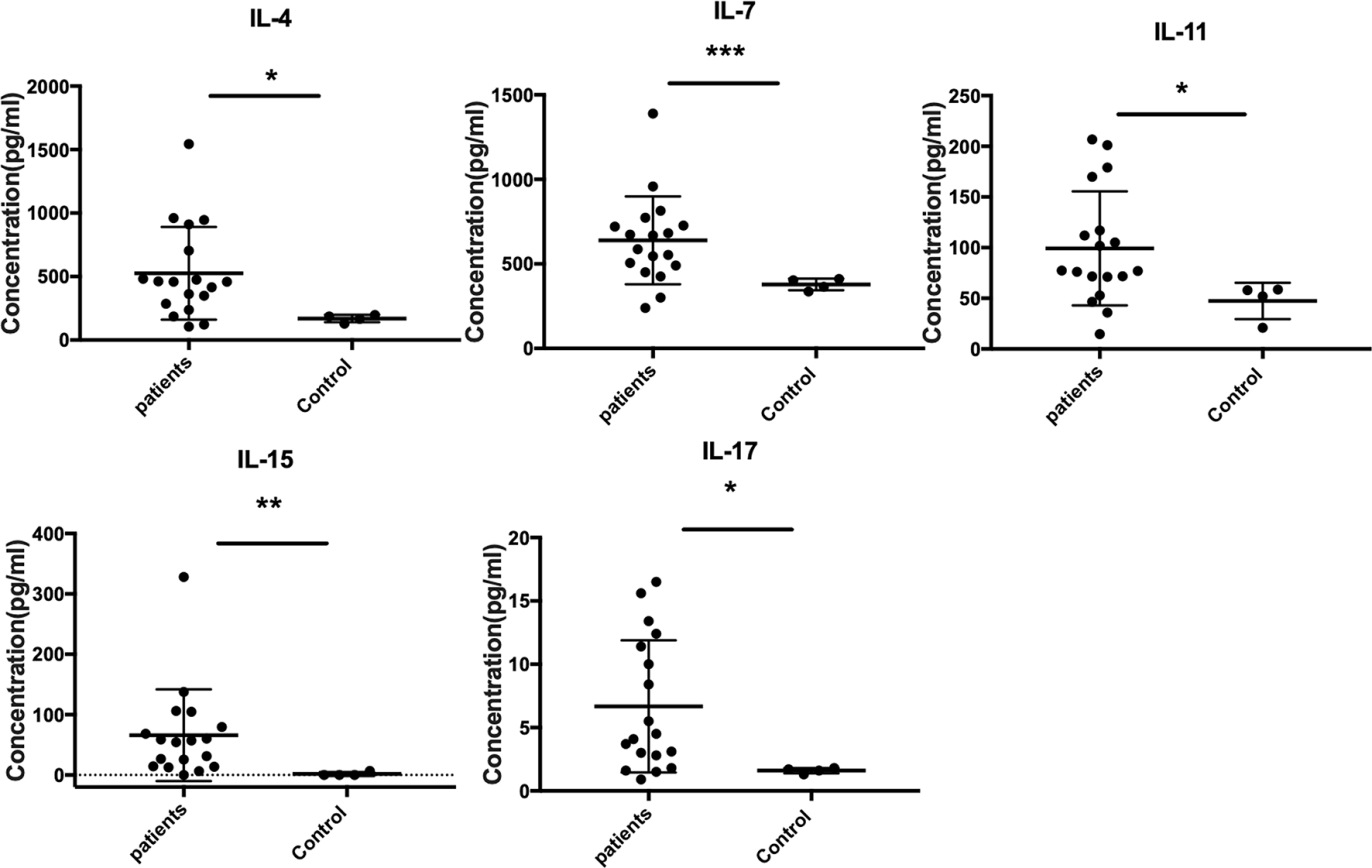
Upregulated expressed cytokines (IL-4, IL-7, IL-15, IL-11, and IL-17) in CBM patients compared to healthy controls (unpaired *t*-test, *p* < 0.05). *P ≤ 0.05, **P ≤ 0.01, ***P ≤ 0.001.

**Figure 3:**
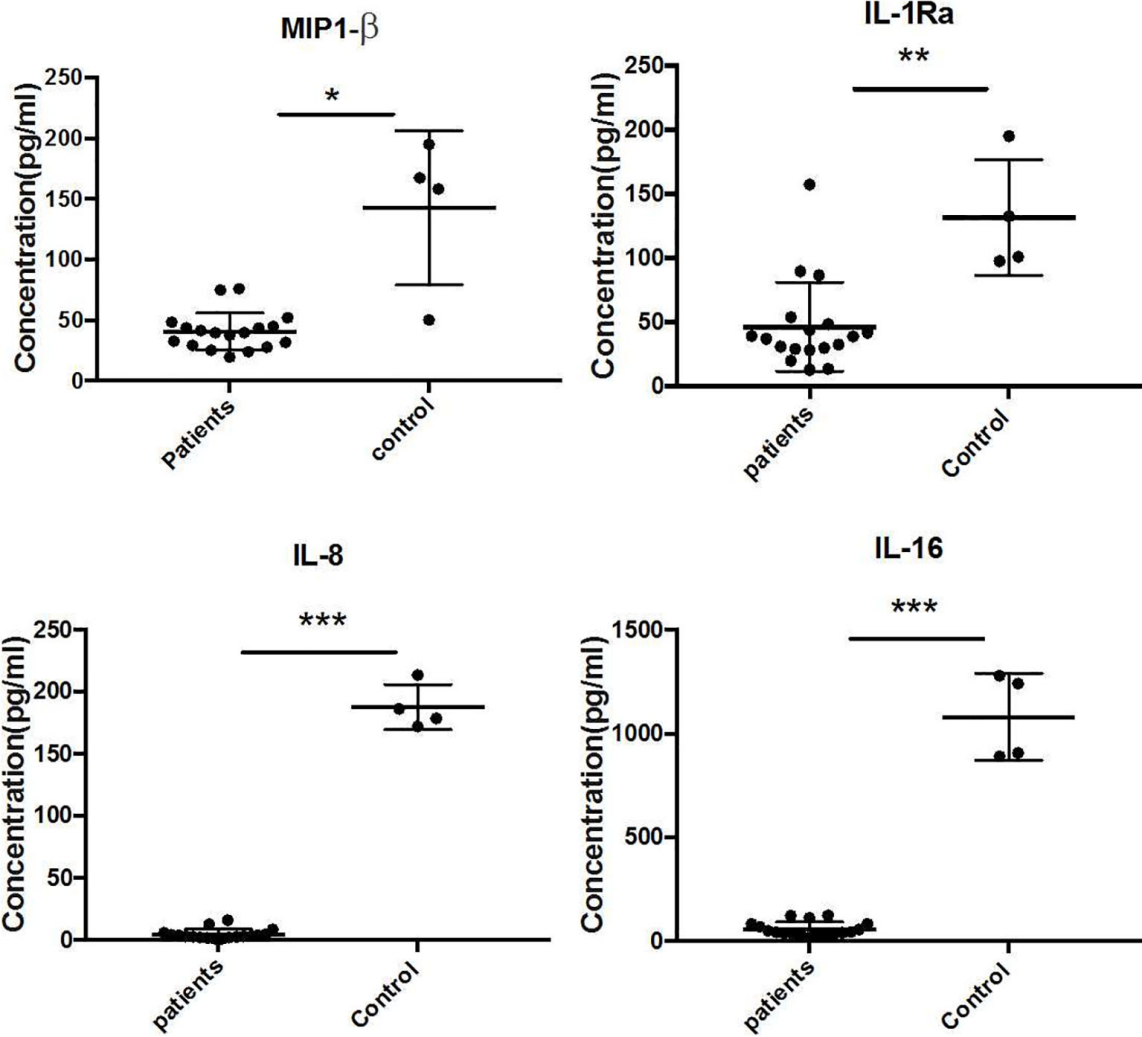
Downregulated expressed cytokines (IL-1Ra, MIP-1β, IL-8, and IL-16) in CBM patients compared to healthy controls (unpaired *t*-test, *p* < 0.05). *P ≤ 0.05, **P ≤ 0.01, ***P ≤ 0.001.

The Quantibody Human Inflammation Array3 was employed to determine 40 human cytokines that are normally presented as an inflammation response of host cells. We compared the expressed level of cytokines between patients and healthy donors. The results indicated that IL-4, IL-7, IL-15, IL-11, and IL-17 were upregulated (**Figure 2**), while IL-1Ra, MIP-1β, IL-8, and IL-16 were downregulated (**Figure 3**) (unpaired *t*-test, *p* < 0.05). We also compared the expression level of cytokines in the patients with severe form and mild form. Thirty-four cytokines were upregulated, while 6 were downregulated between severe and non-severe patients (**Supplementary Figure S1**). IL-4, a marker cytokine of Th2, was highly induced in severe form cases compared to mild form cases (*p* < 0.05). However, the production of other cytokines showed no significant difference between severe and mild form cases (*p* > 0.05).

## Discussion

The age distribution of patients (mean 61.38 ± 11.20 years) in this study was similar to reports from Taiwan (mean 65.9 ± 13.6 years) and Guangdong (59 years, 29 to 83 years) (Fransisca et al., 2017; Yang et al., 2018). The gender ratio (4.6:1) was higher than the report from Taiwan (2.75:1) (Yang et al., 2018) and lower in Guangdong (6.5:1) (Fransisca et al., 2017). The male patients were still the dominant affected population. The skin lesions of most patients (89%) were limited to extremities. The occupation (56% farmers), trauma history (38%), and distribution of lesion sites reemphasize that traumatic inoculation from a contaminated environment is the primary mode of infection in CBM. Fifty-eight percent of patients claimed no underlying diseases, while 8 cases had immunosuppressed diseases including diabetes, systemic lupus erythema, nephrotic syndrome, lung cancer, and chronic colitis. The skin lesion was limited to body sites instead of spreading. We assumed that the local immunity may play an important role to contain the infection. In our study, verrucous form was the most common skin lesion (42%), similar to a report from Taiwan (43%) (Yang et al., 2018). The long duration, bad compliance, and improper therapy may be the main reasons for the high proportion of severe skin lesions (40%). We also supposed long treatment time and expensive medical expense lead to bad compliance, which may be responsible for low healing rate (3 cases) in this cluster of cases.

The combination therapy of oral terbinafine and itraconazole, which was proved to have a synergistic effect against *Fonsecaea* spp. in a previous study (Zhang et al., 2009), was used for the treatment of 31 cases and 68% improved or were cured. This combined therapeutic strategy was supposed to be effective and safe in the management of CBM. Photodynamic therapy as a safe adjunct therapy was also used to treat CBM (Hu et al., 2019; Huang et al., 2019). Three patients who received ALA-PDT improved greatly in 3–4 weeks. However, the high price of the photosensitizer prevented it from being widely used by more patients. In total, 65% of the patients improved or were cured, but we still face the challenges of when will the endpoint of therapy be and how can we reduce relapse.

In Madagascar and Brazil, *F. pedrosoi* is the main causative agent of CBM (Esterre et al., 1996; Silva et al., 1998; Gomes et al., 2016). In Japan, Yaguchi et al. (2007) found 33 isolates previously identified morphologically as *F. pedrosoi* were *F. monophora* actually. In China, there were more than 500 CBM cases reported since 1952. The predominant agent of CBM was *C. carrionii* in Northern China and *F. monophora* in Southern China (Xi et al., 2009). *F. nubica* is a genetic sibling of *F. monophora*. So far, there are more than 40 strains of *F. nubica* reported in published literature from Brazil and China (Gomes et al., 2016; Fransisca et al., 2017; You et al., 2019). Surprisingly, we did not isolate *F. pedrosoi* in the Guangdong region. These results seemed to confirm again that *F. pedrosoi* was found nearly exclusively in Central and South America, while *F. nubica* and *F. monophora* were distributed worldwide (Esterre et al., 1996; Minotto et al., 2001). Interestingly, incidence of *F. nubica* infection was comparable with that of *F. monophora* (17 vs. 28) in this study. Referring to Fransisca’s reports in Guangdong (Fransisca et al., 2017) (15 of 60 cases caused by *F. nubica*), we found an increased ratio of *F. nubica* infection. The major difference was the time Fransisca’s data were collected (before 2015); our data were collected from 2016 to 2021. Whether the increased ratio of *F. nubica* infection resulted from a change of pathogen profile was still unknown. Long-term surveillance and molecular epidemiology study for all CBM cases are greatly needed in the future.

YeastOne^®^ is a widely used commercial antifungal test product, and it shows accuracy and reproducibility for susceptibility of *Candida* (Cuenca-Estrella et al., 2005; Eschenauer et al., 2014), as well as in filamentous fungi, e.g., *Aspergillus*, *Sporothrix*, dermatophytes, and dematiaceous fungi (Pujol et al., 2002; Siopi et al., 2017; Zheng et al., 2019). Compared to CLSI (the National Committee for Clinical Laboratory Standards reference broth microdilution antifungal susceptibility testing method), this method has the advantages of being time-efficient, cost-effective, simple, and easily handled. Zheng et al. (2019) reported that YeastOne^®^ showed high agreement from 85.3% to 100% compared to CLSI in 9 antifungal agents against *F. pedrosoi*. In our study, all the azoles except fluconazole displayed low MICs, indicating that these azoles may have good therapeutic effects on infection caused by *Fonsecaea.* Although voriconazole had a low MIC range (0.015–0.5 μg/ml) compared to Najafzadeh’s report (Najafzadeh et al., 2010), they had similar MIC90 values (0.125 μg/ml vs. 0.25 μg/ml). Most MICs of Azoles obtained were similar to those in other studies of *Fonsecaea* by using CLSI (Najafzadeh et al., 2010). Echinocandins had a limited role in the treatment of CBM for *F*. *monophora* and *F*. *nubica*, which was also observed in other studies (Najafzadeh et al., 2010; Badali et al., 2013). There were no significant differences in either MIC90 or MIC50 between *F. monophora* and *F. nubica* in all tested antifungal agents, which was consistent with archived studies (Najafzadeh et al., 2010; Badali et al., 2013; Coelho et al., 2018). The results of our test of susceptibility to *Fonsecaes* spp. *in vitro* using the yeastone^®^ method had high consistency with other reports using the ClSI method, which confirmed that it was an effective alternative method for determining susceptibility of *Fonsecaea* spp.

Previous studies indicated that severe CBM produced high levels of IL-10 and low levels of IFN-γ together with inefficient T-cell proliferation. Meanwhile, patients with the mild form showed intense production of IFN-γ, low levels of IL-10, and efficient T-cell proliferation (Mazo et al., 2005). In our study, we only found that IL-4 was highly produced in severe compared to mild form cases (*p* < 0.05). However, no significant differences were found in cytokines IL-10, IFN-γ, and TNF-alpha comparing severe form to mild form cases, as well as in patients and healthy controls. In addition, we found that IL-7 and IL-15 (Th1 cytokines), IL-11 (proinflammatory cytokine), and IL-17 (Th17 cytokine) significantly increased comparing patients to healthy controls (*p* < 0.05), but there was no significant difference when comparing severe to mild form cases (*p* > 0.05). The antifungal cytokines—Type1 (IL-7 and IL-15), Th17 (IL-7), and proinflammatory (IL-11)—were all significantly enhanced in CBM patients, presumably as a compensatory response to the long-term fungal infection. Surprisingly, we also detected the significantly reduced expression of IL-1Ra, MIP-1β, IL-8, and IL-16 in the serum of CBM patients. The cytokines of MIP-1β, IL-8, and IL-16 played important roles in recruiting macrophages, neutrophils, and CD4 molecules, respectively, which are crucial immune cells against fungal infection (Cruikshank et al., 1994; Maurer and von Stebut, 2004; Remick, 2005). Downregulation of these cytokines indicated impaired immune response to eliminate fungus in CBM cases, which probably contributed to chronic duration of CBM. IL-1Ra (IL-1 receptor antagonist) was a cytokine to inhibit activities of IL-1A, IL-1B. IL-1Ra knock out mice were highly resistant to invasive aspergillosis (Gresnigt et al., 2014), which seemed to be paradoxical to the reduced expression of IL-1Ra in CBM patients. The role that IL-1Ra played in *Fonsecaea* infection still needs further investigation. In this study, we obtained the different cytokine profiles compared to previous studies. The probable explanation was the sample types we used in our study differ from the peripheral blood mononuclear cells (PBMCs) used in archived studies (Mazo et al., 2005). Therefore, future studies will focus on these differently expressed cytokines in both blood and tissues of CBM patients to address the remaining questions.

In conclusion, our study showed a comprehensive clinical and molecular characteristic of a cluster of 45 CBM cases in Guangdong, China, which may represent the regional trends in this subtropical hyper-endemic area of CBM. These data will definitely contribute to better management and clinical therapy, and will help countries still struggling to combat this difficult fungal infection in the West Pacific.

## Data Availability Statement

The datasets presented in this study can be found in online repositories. The names of the repository/repositories and accession number(s) can be found in the article/**Supplementary Material**.

## Ethics Statement

The studies involving human participants were reviewed and approved by the Ethics Committee Board of Dermatology Hospital of Southern Medical University. The patients/participants provided their written informed consent to participate in this study.

## Author Contributions

HL and JS designed the experiments and analyzed the data. HL wrote the manuscript. ML, WC, YC, YL, HH, ZX, and WZ performed the experiments and collected the clinical data. HL and JS are the first co-authors and contributed equally to this article. All works were performed under the guidance of LX. All authors contributed to the article and approved the submitted version.

## Funding

This study was kindly supported by grants from the National Natural Science Foundation of China (81601746 and 81873960).

## Conflict of Interest

The authors declare that the research was conducted in the absence of any commercial or financial relationships that could be construed as a potential conflict of interest.

## Publisher’s Note

All claims expressed in this article are solely those of the authors and do not necessarily represent those of their affiliated organizations, or those of the publisher, the editors and the reviewers. Any product that may be evaluated in this article, or claim that may be made by its manufacturer, is not guaranteed or endorsed by the publisher.

## Supplementary Material

Supplementary Figure S1 The comparison of each cytokines between severe vs mild group, and tested by T test. The p value (-log_10_ P) and fold change (FC) (log_2_ FC), were plot indicated the cytokines with statistical support between these two groups. Purple dot indicated the different makers between two groups with statistical support.
