# Supplementary figures and images for: Molecular Characteristics of Regional Chromoblastomycosis in Guangdong, China: Epidemiological, Clinical, Antifungal Susceptibility, and Serum Cytokine Profiles of 45 Cases
Source: Front Cell Infect Microbiol. 2022 Feb 18;12:810604. doi: 10.3389/fcimb.2022.810604 (PMC8894709; doi:10.3389/fcimb.2022.810604)

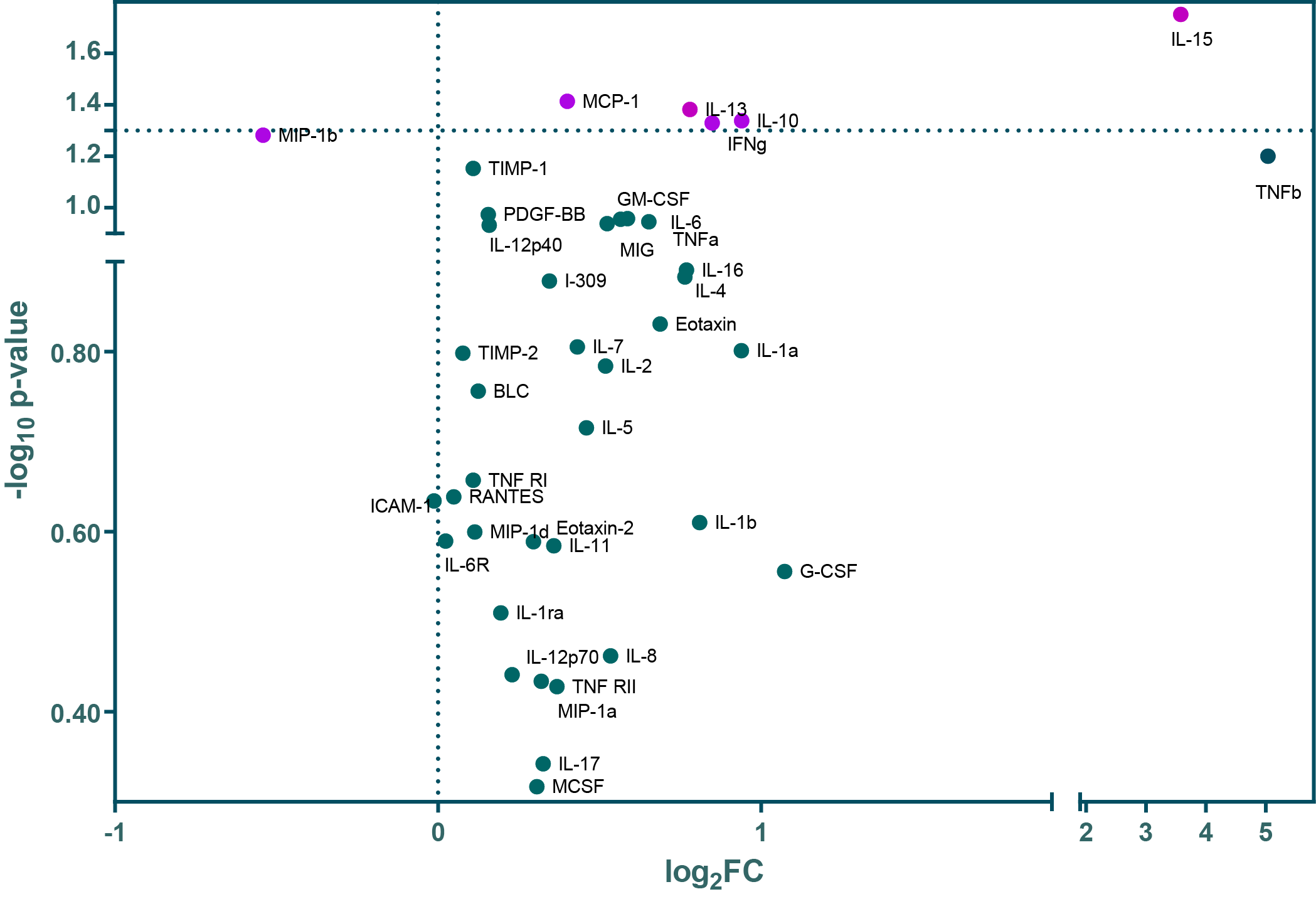

Supplement: Supplementary Figure S1 — The comparison of each cytokines between severe vs mild group, and tested by T test. The p value (-log10 P) and fold change (FC) (log2 FC), were plot indicated the cytokines with statistical support between these two groups. Purple dot indicated the different makers between two groups with statistical support. [file Image_1.tif]
