# Supplementary material for: Molecular Characteristics of Regional Chromoblastomycosis in Guangdong, China: Epidemiological, Clinical, Antifungal Susceptibility, and Serum Cytokine Profiles of 45 Cases
Source: Front Cell Infect Microbiol. 2022 Feb 18;12:810604. doi: 10.3389/fcimb.2022.810604 (PMC8894709; doi:10.3389/fcimb.2022.810604)
Supplement: Supplementary file 2 [file Table_1.doc]

Table S1. Clinical Data of 45 cases of Chromoblastomycosis.

| **N** | **Strain number** | **species** | **sex** | **Age(y)** | **Occupation** | **Trauma** | **Underlying disease** | **Time of evolution** | **Sites of involvement** | **severity** | **Lesion type** | **Treatment** | **Treatmenttime** | **Outcome** |
| --- | --- | --- | --- | --- | --- | --- | --- | --- | --- | --- | --- | --- | --- | --- |
| 1 | SMUD001 | *F.m.* | F | 67 | Farmer | N | Diabetes | 6m | Right forearm | Mild | Verrucous | ITR+TER  +Surgery | 6 | Cure |
| 2 | SMUD002 | *F.n.* | M | 68 | NA | Y | N | 4Y | Right forearm | Moderate | Plaque | ITR+TER | 18m | Relapse |
| 3 | SMUD003 | *F.m.* | M | 55 | NA | NA | NA | 4Y | Right forearm | Mild | Plaque | TER | NA | Lose follow-up |
| 4 | SMUD004 | *F.m.* | M | 49 | NA | N | N | 6Y | Left lower extremity | Moderate | Plaque | ITR+TER | 3m | Improve |
| 5 | SMUD005 | *F.m.* | F | 51 | NA | NA | NA | 2Y | Right forearm | Mild | Cicatricial | ITR+TER | 16m | Relapse |
| 6 | SMUD006 | *F.n.* | M | 57 | Farmer | Y | N | 10Y | Left arm | Severe | Verrucous | ITR+TER | NA | Lose follow-up |
| 7 | SMUD032 | *F.m.* | M | 74 | NA | NA | Na | 9Y | Left wrist | Moderate | Plaque | ITR+TER | 5m | Improve |
| 8 | SMUD008 | *F.n.* | M | 62 | Farmer | Y | N | 15Y | Right lower extremity | Severe | Verrucous | ITR+TER  +Thermal therapy | 8m | Improve |
| 9 | SMUD009 | *F.n.* | M | 59 | NA | NA | N | 8m | Abdomen | Moderate | Plaque | ITR | 14m | Improve |
| 10 | SMUD010 | *F.n.* | F | 66 | Farmer | N | Coronary Heart Disease | 3m | Left buttock | Severe | Plaque | ITR+TER | NA | Lose follow-up |
| 11 | SMUD037 | *F.m.* | M | 52 | Farmer | Y | Hyperlipemia | 6m | Left ankle | Mild | Plaque | ITR+TER  +PDT | 3m | Cure |
| 12 | SMUD013 | *F.m.* | M | 68 | Farmer | N | Hypertension | 7Y | Right arm | Severe | Plaque | ITR+TER | 2m | Improve |
| 13 | SMUD017 | *F.n.* | M | 64 | NA | N | N | 20Y | Right foot and right lower extremity | Severe | Mixed form | ITR+TER  +PDT | 30m | Improve |
| 14 | SMUD020 | *F.m.* | M | 24 | No work | Y | SLE | 3Y | Right foot and right lower extremity | Moderate | Nodular | ITR+TER | 4m | Improve |
| 15 | SMUD036 | *F.n.* | M | 52 | Farmer | N | N | 20Y | Left knee | Severe | Verrucous | ITR+TER | 3m | Improve |
| 16 | SMUD034 | *F.m.* | F | 65 | Worker | N | N | 6Y | Left lower extremity | Mild | Plaque | ITR | 2m | Improve |
| 17 | SMUD023 | *F.n.* | M | 77 | Teacher | Y | N | 25Y | Left foot and Left lower extremity | Severe | Tumorous | ITR+TER | 24m | Improve |
| 18 | SMUD024 | *F.n.* | M | 53 | Mill worker | N | N | 2Y | Right lower extremity | Mild | Cicatricial | No therapy | N | Lose follow-up |
| 19 | SMUD025 | *F.n.* | M | 60 | Farmer | N | N | 20Y | Left foot | Severe | Verrucous | ITR+TER | 12m | Improve |
| 20 | SMUD026 | *F.m.* | F | 72 | Farmer | N | N | 3Y | Right lower extremity | Severe | Verrucous | ITR+TER | 3m | Improve |
| 21 | SMUD033 | *F.m.* | M | 72 | NA | Y | NA | 20Y | Left lower extremity and left foot | Severe | Verrucous | ITR+TER (intermittent) | 48m | Relapse |
| 22 | SMUD029 | *F.n.* | M | 62 | No work | N | N | 1Y | Left lower extremity | Moderate | Verrucous | ITR+ surgery | 5m | Improve |
| 23 | SMUD030 | *F.m.* | M | 64 | NA | NA | NA | 3Y | Right forearm | Mild | Verrucous | ITR | NA | Lose follow-up |
| 24 | SMUD031 | *F.n.* | M | 59 | Farmer | NA | N | 11Y | Right forearm | Severe | Verrucous | No therapy | N | Lose follow-up |
| 25 | SMUD035 | *F.m.* | F | 54 | Farmer | NA | N | 2Y | Left arm | Mild | Verrucous | ITR+TER | 5m | Improve |
| 26 | SMUD051 | *F.n.* | M | 42 | Farmer | Y | N | 3m | Left waist | Mild | Plaque | ITR+TER  +Surgery | 4m | Cure |
| 27 、 | SMUD049 | *F.m.* | M | 65 | Farmer | N | N | 4Y | Right arm | Moderate | Verrucous | ITR | 1m | Relapse |
| 28 | SMUD041 | *F.m.* | M | 77 | No work | Y | Diabetes | 6Y | Left hand | Severe | Verrucous | ITR+TER | 5m | Improve |
| 29 | SMUD050 | *F.m.* | M | 86 | No work | NA | NA | 3Y | Left hand | Mild | Verrucous | ITR+TER | 1m | Lose follow-up |
| 30 | SMUD048 | *F.m.* | M | 60 | Farmer | N | N | 30Y | Back | Severe | Mixed form | ITR+TER | 1m | Lose follow-up |
| 31 | SMUD047 | *F.n.* | M | 61 | Farmer | Y | N | 2Y | Right foot | Moderate | Verrucous | No therapy | NA | Lose follow-up |
| 32 | SMUD043 | *F.n.* | M | 69 | Farmer | Y | N | 2Y | Left knee | Moderate | Plaque | ITR | 11m | Improve |
| 33 | SMUD039 | *F.m.* | F | 56 | Farmer | N | N | 5y | Right wrist | Mild | Plaque | ITR+TER | 5m | Lose follow-up |
| 34 | SMUD044 | *F.m.* | M | 66 | Farmer | N | Diabetes | 15Y | Left upper extremity | Severe | Mixed form | ITR+TER | 37m | Improve |
| 35 | SMUD040 | *F.m.* | M | 65 | Farmer | Y | Hypertension | 10Y | Left hand and right hand | Severe | Mixed form | ITR+ Surgery | 5m | Improve |
| 36 | SMUD045 | *F.m.* | M | 34 | Worker | N | N | 5Y | Right lower extremity | Severe | Verrucous | ITR+TER | 1m | Relapse |
| 37 | SMUD052 | *F.m.* | M | 68 | Farmer | Y | N | 10Y | Left lower extremity | Severe | Verrucous | ITR+TER  +Surgery | 5m | Improve |
| 38 | SMUD055 | *F.m.* | M | 57 | NA | NA | Chronic colitis and bronchitis | 2Y | Left lower extremity | Moderate | Plaque | ITR | 1m | Lose follow-up |
| 39 | SMUD054 | *F.m.* | M | 67 | No work | N | Lung cancer | 10Y | Left wrist | Moderate | Plaque | ITR+TER | 4m | Improve |
| 40 | SMUD053 | *F.m.* | M | 72 | Farmer | Y | N | 8m | Left hand | Mild | Verrucous | ITR+TER | 1m | Improve |
| 41 | SMUD046 | *F.m.* | M | 51 | Worker | N | N | 6m | Back | Severe | Cicatricial | ITR+TER | 5m | Improve |
| 42 | SMUD042 | *F.n.* | M | 74 | Farmer | Y | Cerebral artery stenosis | 10Y | Left foot | Moderate | Plaque | ITR+TER | 1m | Improve |
| 43 | SMUD038 | *F.n.* | M | 70 | Farmer | N | N | 5Y | Right arm | Moderate | Plaque | ITR+TER  +PDT | 8m | Improve |
| 44 | SMUD056 | *F.m.* | M | 59 | Farmer | Y | SLE | 1Y | Right arm, Left hand | Moderate | Plaque | ITR | 2m | Improve |
| 45 | SMUD057 | *F.m.* | F | 57 | Farmer | Y | Nephrotic syndrome | 3y | Left hand | Mild | Verrucous | ITR+ Surgery | 1m | Improve |

Y: Yes, N: No, NA : Not available, M: male, F: female，SLE：Systemic lupus erythematosus，ITR: itraconazole, TER: terbinafine, PDT: Photodynamic therapy.
