# Supplementary material for: Molecular Characteristics of Regional Chromoblastomycosis in Guangdong, China: Epidemiological, Clinical, Antifungal Susceptibility, and Serum Cytokine Profiles of 45 Cases
Source: Front Cell Infect Microbiol. 2022 Feb 18;12:810604. doi: 10.3389/fcimb.2022.810604 (PMC8894709; doi:10.3389/fcimb.2022.810604)
Supplement: Supplementary file 3 [file Table_2.docx]

**Table S2**  Genbank accession number of 45 clinical strains

| **N** | **Strain number** | **Genebank accession**  **number** |
| --- | --- | --- |
| 1 | SMUD001 | MN960516 |
| 2 | SMUD002 | MN960517 |
| 3 | SMUD003 | MN960518 |
| 4 | SMUD004 | MN960519 |
| 5 | SMUD005 | MN960520 |
| 6 | SMUD006 | MN960521 |
| 7 | SMUD032 | MN960535 |
| 8 | SMUD008 | MN960522 |
| 9 | SMUD009 | MN960523 |
| 10 | SMUD010 | MN960524 |
| 11 | SMUD037 | MN960540 |
| 12 | SMUD013 | MN960525 |
| 13 | SMUD017 | MN960526 |
| 14 | SMUD020 | MN960527 |
| 15 | SMUD036 | MN960539 |
| 16 | SMUD034 | MN960537 |
| 17 | SMUD023 | MN960528 |
| 18 | SMUD024 | MN960529 |
| 19 | SMUD025 | MN960530 |
| 20 | SMUD026 | MN960531 |
| 21 | SMUD033 | MN960536 |
| 22 | SMUD029 | MN960532 |
| 23 | SMUD030 | MN960533 |
| 24 | SMUD031 | MN960534 |
| 25 | SMUD035 | MN960538 |
| 26 | SMUD051 | OK605594 |
| 27 | SMUD049 | OK605592 |
| 28 | SMUD041 | OK606065 |
| 29 | SMUD050 | OK605590 |
| 30 | SMUD048 | OK606051 |
| 31 | SMUD047 | OK606063 |
| 32 | SMUD043 | OK606006 |
| 33 | SMUD039 | OK606005 |
| 34 | SMUD044 | OK606052 |
| 35 | SMUD040 | OK606056 |
| 36 | SMUD045 | OK606062 |
| 37 | SMUD052 | OK605595 |
| 38 | SMUD055 | OK605596 |
| 39 | SMUD054 | OK605593 |
| 40 | SMUD053 | OL440973 |
| 41 | SMUD046 | OK606003 |
| 42 | SMUD042 | OK606007 |
| 43 | SMUD038 | OL440974 |
| 44 | SMUD056 | OK605584 |
| 45 | SMUD057 | OK605559 |
